# A qualitative study of assistant nurses' experiences of palliative care in residential care

**DOI:** 10.1002/nop2.159

**Published:** 2018-05-29

**Authors:** Camilla Udo, Maria Neljesjö, Ingegerd Strömkvist, Marie Elf

**Affiliations:** ^1^ School of Education, Health and Social Studies Dalarna University Falun Sweden; ^2^ Center for Clinical Research Dalarna Falun Sweden; ^3^ Karolinska Institutet Department of Neurobiology, Care Sciences and Society Stockholm Sweden; ^4^ Chalmers University of Technology School of Architecture Gothenburg Sweden

**Keywords:** assistant nurses, critical incident technique, organization, palliative care, Sweden

## Abstract

**Aim:**

To explore assistant nurses' experiences and perceptions of both positive and negative aspects of providing palliative care for older people in residential care facilities.

**Design:**

A qualitative explorative study.

**Methods:**

Critical incidents were collected through semi‐structured face‐to‐face interviews and analysed by performing a qualitative content analysis.

**Results:**

A total of 40 critical incidents from daily work was described by assistant nurses. The results showed that close cooperation between unlicensed and licensed professionals was crucial to provide good care but was sometimes negatively affected by the organizational structure. The availability of professionals was identified as a critical factor in providing good care at the end of life in a consultative organization. The most prominent findings were those that indicated that, especially in a consultative organization, there seems to be a need for clear roles, comprehensive and clear care plans and a solid support structure to ensure continuity of care.

## 1 INTRODUCTION

In the Western world, the need for palliative care for older people is currently increasing due to the ageing population living with chronic illnesses (World Health Organization [WHO] 2011). The number of older people with frail health in need of palliative care either in their home or in residential care facilities (RCFs) is rising (Abarshi et al., 2009; Penders, van der Block, Donker, Deliens, & Onwuteaka‐Philpsen, 2015). This need places huge demands on healthcare organizations and on each care provider. The staff must have sufficient skills and knowledge about both palliative care and interacting with the patient and the patient's family, such as inviting family members into the care process (WHO, 2011). In Swedish RCFs, the organization of services has changed during the last few decades and the number of registered nurses (RNs) has decreased, which has led to RNs often working on a consulting basis among several RCFs (Josefsson, 2009). Daily care, including personal care and practical support, is therefore often provided by assistant nurses (ANs), also known as nurse aids or care assistants, who are unlicensed professionals (Beck, Jakobsson, & Edberg, 2014; Beck, Törnquist, & Edberg, 2014; National Board of Health and Welfare, 2012). This type of organization requires clear roles and well‐functioning coordination mechanisms (Karacsony, Chang, Johnson, Good, & Edenborugh, 2015). Because palliative care situations are often complex, it is important to explore the experiences and perceptions of ANs about both the positive and negative aspects of providing palliative care for older people in RCFs.

## 2 BACKGROUND

Palliative care philosophy has had an impact on Swedish health care since the 1980s (Beck‐Friis, 2008). Swedish laws emphasize the importance of promoting health, alleviating suffering and facilitating a peaceful and dignified environment at the end of a person's life irrespective of cultural background, age, sex or social conditions (SFS 1982: 763, 2001: 453). The goal of palliative care is to offer a person‐centred approach that stresses each patient's right to be involved in the care process and to make personal choices (World Health Organization, 2011). According to the World Health Organization (2011), palliative care affirms life and regards dying as a normal process where death is neither hastened nor postponed and is a human right to which everyone should have access. In palliative care, physical, psychosocial and existential dimensions are acknowledged (World Health Organization, 2011), which means that healthcare services need to integrate several dimensions into palliative care situations so that the patient is considered a whole and unique person (Saunders, 1978). It is also essential to introduce palliative care early during the process (Hui & Bruera, 2016). The World Health Organization has called for initiatives to improve palliative care for older people (2011).

The care given during the palliative phase is important not only for the patient but also for the patient's family. Supporting the patient's family in understanding and handling their experiences and reactions is an important part of palliative care (Carlander, Sahlberg‐Blom, Hellström, & Ternestedt, 2011; Linderholm & Friedrichsen, 2010; Wetle, Shield, Teno, Miller, & Welch, 2005; World Health Organization, 2011). The family may also need support to be prepared for the death of their relative (Kim, Carver, Spiegel, Mitchell, & Cannady, 2015). Goodridge, Bond, Cameron, and McKean (2005) showed that coordinated care has a profound impact on how the dying process is experienced by both the patient and the family as well as by the professionals involved in care. Other studies have shown that when there is a certain amount of planning and routine and when professionals feel confident in their roles, good palliative care can be achieved (Stillman, Strumpf, Capezuti, & Tuch, 2005). Moreover, the absence of teamwork has been described as a significant barrier to good care (Hanson, Henderson, & Menon, 2002).

Swedish RCFs are provided by the municipalities and offer services (e.g. cleaning, laundry service and meals) and care 24 hr a day (Fukushima, Adami, & Palme, 2010). The older person rents an apartment in an RCF. For the past 5 years, there has been a substantial decrease in the number of RCFs for older people and only those in very frail health are entitled to a place in such facility (Håkansson, Öhlén, Morin, & Cohen, 2014; National Board of Health and Welfare, 2005). Consequently, most people living in RCFs have high levels of physical and cognitive disabilities and usually need help with personal care (Bravell, Malmberg, & Berg, 2010). Research has shown that a significant percentage of older people die relatively shortly after moving into an RCF (Andersson, Hallberg, & Edberg, 2007).

In Sweden, bedside care (also the provision of palliative care) for older people in RCFs is provided by ANs who often have 2 years of secondary education and training without specific nursing or palliative care education (National Board of Health and Welfare, 2016). ANs are expected to support the RN, who has the main responsibility of the care. ANs are taking care of the resident, such as assisting the older person with personal hygiene, oral care and medications. RNs can also delegate more advanced care tasks to ANs, such as giving insulin, treating wounds and rinsing urine catheters. Often, RNs delegate the responsibility of giving medication to ANs when needed, such as when older patients are in pain. Several studies have underlined the need to improve the education of ANs in palliative care, particularly in the RCF setting (Eriksson, Bergstedt, & Melin‐Johansson, 2015; Reimer‐Kirkham, Sawatzky, Roberts, Cochrane, & Stajduhar, 2016; Ronaldson, Hayes, Carey, & Aggar, 2008), as one way to strengthen the professional care given by ANs (Daly & Szebehely, 2012). The National Board of Health and Welfare (2011) emphasized that staff involved in elderly care must be skilled and experienced. However, there are large differences in educational levels among staff in Swedish RCFs (National Board of Health and Welfare, 2011), with the majority having only secondary education or less in nursing care.

Research has shown that although ANs attempt to perform their best, they often find it difficult to integrate guidelines into practice, struggling between following mandatory guidelines and making time to meet the residents' needs (Åhlin, Ericson‐Lidman, Norberg, & Strandberg, 2014) and incorporating certain aspects, such as existential issues, into the caring process (Beck, Törnquist, Broström, & Edberg, 2012; Beck, Jakobsson, et al., 2014; Dwyer, Andershed, Nordenfelt, & Ternestedt, 2009). Often, ANs are more focused on tasks supporting the person's physical needs (Beck et al., 2012; Beck, Jakobsson, et al., 2014; Dwyer et al., 2009). Furthermore, the findings by Dwyer et al. (2009) highlight a conflict between the ethical care principles and the care delivered by ANs. This type of conflict often involves inner dilemmas that may increase perceived stress (Udo, Danielson, Henoch, & Melin‐Johansson, 2014) and are related to the staff's mental exhaustion (Gustafsson & Szebehely, 2005).

To achieve high qualitative palliative care, it is necessary to develop strategies for communication between the staff and to clarify the roles of those involved in care (Reimer‐Kirkham et al., 2016). Implementing and integrating palliative care must be context‐sensitive and supported by the leadership to succeed (Pesut et al., 2014; Reimer‐Kirkham et al., 2015, 2016). In a survey conducted among RNs and ANs in British Columbia (Sawatzky, Roberts, Reimer‐Kirkham, & Stajduhar, 2014), the authors emphasized the importance of integration and contextualization in the specific healthcare system when identifying and bridging gaps in palliative care. Thus, to achieve high‐quality palliative care and to give adequate support to the ANs, it is important to consider the structure of the organization and to assess how care is provided in an RCF. It is of utmost importance to study how the ANs closest to the patient perceive and experience the care. Most research has focused on RNs' role in palliative care, but there is a lack of research exploring the role of ANs in care. Since ANs are the ones providing daily and end‐of‐life care, it is important to explore palliative care from their perspective. This study will contribute to a greater understanding of ANs' role in palliative care, which in turn will contribute to increased insight into palliative care in the context of RCF.

## 3 THE STUDY

### 3.1 Design

This was an explorative qualitative study where semi‐structured interviews were conducted to collect data on actual care situations through critical incidents (Flanagan, 1954; Kempainen, 2000). Data were analysed via a qualitative content analysis (Graneheim & Lundman, 2004).

### 3.2 Settings and participants

This study was conducted in two municipalities of central Sweden with populations of 20,000 and 15,000, respectively. A purposeful sample (Creswell, 2009) of eight ANs working in residential care was obtained with the help of unit managers and head nurses who based their recommendations on the following three inclusion criteria: ANs with at least 1 year of experience in residential care of various ages who would be available for an interview during the spring (March–May) of 2012. Experience in delivering palliative care was not explicitly requested since caring for patients in need of palliative care is unavoidable when working in RCFs.

### 3.3 Procedure

In both municipalities, the managers of all RCFs were contacted by telephone and e‐mail and were provided oral and written information about the study. When the managers gave their permission and signed an ethical agreement, they collaborated with the head nurse of the two municipalities to suggest one eligible RCF from each community and ANs who met the inclusion criteria and who were available at a given time. These ANs were then contacted first by e‐mail and then in person by the researchers who informed the ANs of the study. The ANs who expressed interest in participating in the study were given a few days to decide before they were contacted again by telephone to determine whether they were still interested in participation. The time and location of the interview were then scheduled.

### 3.4 Data collection

Data were collected in the spring of 2012 through eight face‐to‐face interviews outlined using a semi‐structured interview guide (Kvale, 2006). The interviews ranged in length from 20‐50 min and were tape‐recorded and transcribed verbatim. During the interviews, the participants were asked to describe their experiences and perceptions of providing palliative care for older people in residential care. If they had not already spontaneously done so, they were then asked to specifically describe critical incidents they had experienced while caring for a dying patient, namely, one incident that had a positive effect on the palliative care situation and one that had a negative effect. Through the critical incident technique (CIT), real care situations in practical settings were described based on the ANs' subjective perceptions (Flanagan, 1954; Kempainen, 2000). Although critical incidents were originally collected by observations (Flanagan, 1954), the CIT has since been used differently in nursing research. For instance, incidents may be gathered through interviews (Kempainen, 2000) while still focusing on actual defined events. Because the CIT offers a opportunity to focus on actual rather than hypothetical events, the method was considered suitable for the aim of this study.

### 3.5 Data analysis

A qualitative content analysis informed by the methods of by Graneheim and Lundman (2004) was conducted. All interviews were first thoroughly read by the two authors who conducted and transcribed the interviews. The interviews were then combined into a single text and read as a whole by all authors to obtain an overall understanding of the text. From the text, units of meaning were identified before they were condensed and coded. To avoid bias, the steps in the analysis were continuously discussed among the authors before categories were agreed on. The continuous discussion helped to redefine categories to more accurately reflect the data and to make them as transparent and trustworthy as possible.

### 3.6 Ethics

The study was approved by the ethical university committee in the county where the study occurred (DUC 2011/799/90). All participants signed consent forms to participate in the study after they had received oral and written information. Confidentiality was guaranteed, and participants were informed that they could withdraw from the study at any time without any repercussions. Additionally, participants were given the opportunity to stay and discuss their thoughts and emotions if they felt the need for further reflection after the interview had ended.

## 4 RESULTS

The participants were all Scandinavian women, 34–62 (median = 50) years of age with 2 years of postsecondary education and 16–30 (median = 21) years of working experience in RCFs. None of the participants reported any prior specific palliative care education or training. The participants' roles consisted mainly of being present during patients' daily care routines and to support them in these activities.

Using the CIT, the participants described 40 critical incidents from their day‐to‐day practice, with an emphasis on both the positive and negative aspects of providing palliative care for older people in RCFs. The analysis resulted in the identification of three categories: (i) being part of a team; (ii) the presence and provision of psychosocial support; and (iii) the care environment as an important factor in palliative care. These categories are presented with quotations in Table 1.

**Table 1:** Categories and examples of quotations

Categories	Examples of quotations
Being part of a team	“He had four children, and there was always one of the children there with him. And they were all, shall we say, well aware of what would happen. They were calm, which meant that we were calm.” (Interview 7)“There was a man who was very sick, and many of his family members who were with him were very worried. However, our RN had so much to do and went back and forth and could not stay…I wish we could have cooperated more…” (Interview 5)
Presence and provision of psychosocial support	“I sat there by her side…Her relatives had not come yet. And then she just closed her eyes and smiled. It was so nice to just sit there, hold her hand and stroke her gently on the cheek…” (Interview 4)“We all felt really bad. Most of all, one of the younger colleagues who herself had been diagnosed with cancer before…She was completely devastated. It affected us all…” (Interview 1)
The care environment as an important factor in palliative care	“I sat with him, and there was a picture in there that he could see from where he was lying. He looked at it, and he began to tell me…” (Interview 3)“At times, there were two people sleeping in there, which made the room crowded. So when we, the staff, came into the room at night to see that everything was alright, we almost stepped over them.” (Interview 2)

### 4.1 Being part of a team

The participants reported that incidents with a positive impact on palliative care situations included successful cooperation with both the patient's family members and colleagues and support from colleagues (Table 1). The participants described the importance of working towards the same goal based on a care plan. The RN was mentioned as the most important colleague on whom they felt dependent. When the entire care process ran smoothly, the participants perceived this experience as a contribution to safe and dignified palliative care. The participants indicated that they wished for more interdisciplinary teamwork that also included the family and they especially asked for closer teamwork with the RN. Incidents described as having a negative influence on the palliative care situation involved unsuccessful cooperation among different professionals or a lack of a comprehensive and clear care plan. The participants stated that they experienced difficulties when they felt that they were left with too much responsibility without support. Participants stated that if they did not call the RN in time, they were often the individuals who had to motivate care decisions, answer questions and face possible discontent from family members. Other incidents described as having a negative impact on care included a disorganized care process due to a lack of planning, the perception that the RNs lacked expertise or experience and the lack of familiarity of the RN with the dying patient, making it difficult to give adequate care.

### 4.2 Presence and provision of psychosocial support

Participants mentioned that positive incidents involved situations where they had been able to provide family members and the patient with comfort and support and situations where family members were invited and encouraged to stay with the dying patient until death (Table 1). Allowing the family to be present until the time of death was considered a challenge, but the participants viewed this task as important and in line with their own care intentions and ambitions. Participants described negative incidents that involved situations when an older person's wishes were difficult to fully respect or when an older person's wishes went against what the participant thought was right, thus making it difficult to give psychosocial support. For example, when a patient did not wish for the family to be informed about imminent death, the participants were caught in an ethical dilemma. Other negative incidents occurred when medical symptom relief could only be given when prescribed by an RN or a physician, which meant that the participants themselves did not have control over medication administration. Instead, they had to trust others to give relief, although they themselves were the individuals in the room with the patient. When a patient's symptoms were not relieved, their interactions with the professionals and interactions between the professionals and the family were described by the participants as complicated due to anxiety, disappointment and distrust. Psychosocial support was described by the participants as more complex and demanding when the patient reminded the participants of themselves or someone close to them because it led them to become too personally involved as they identified with the dying patient. The participants stated that becoming too involved at a personal and emotional level made it difficult for them to stay present with the dying patient.

### 4.3 The care environment as an important factor in palliative care

Participants noted that positive incidents involved the discovery of how different environmental factors, such as a painting or photography, could awaken the dying person's memories and thus initiate a deeper dialogue (Table 1). The participants expressed that this situation gave opportunities for meaningful dialogues about things that mattered to the dying person. The participants also described experiences of negative incidents about the care environment that affected interactions with the patient and family. For example, a noisy environment and a lack of peace and quiet contributed to the unease and worry of the dying person. The participants explained that if the dying person was spending his or her last days in a residential care home where there were also people with dementia who strayed into the room of the dying, the dying person's peace was disturbed, which hindered conversations and consequently inhibited the provision of good palliative care. A telephone ringing was another example of a disruptive environmental element that was identified as negative in the participants' descriptions of critical incidents. Rooms in RCFs that were too small, which made it difficult for family members to sleep over, were another environmental factor that, according to the participants, created a barrier in providing high‐quality palliative care.

## 5 DISCUSSION

In this study, ANs' experiences and perceptions of palliative care were investigated using the retrospective CIT where participants described 40 critical incidents from their palliative care experience. This study found that ANs believed that interdisciplinary teamwork and support, especially from RNs, was crucial for a good experience in palliative care situations. In addition, the ANs' ability to attend to and give psychosocial support to the patient and family was important. Furthermore, ANs stated that the care environment was a factor affecting palliative care. The analysis showed that either the organization or the environment was optimally designed for a given level of high‐quality palliative care despite the large number of older persons who die in RCFs. This is a serious issue because it might act as a barrier to evidence‐based palliative care in RCFs.

The first category, “being part of a team,” clearly showed that the team was important for ANs' experience of providing good palliative care. It was apparent that ANs played an important role in delivering palliative care to the patient and family despite lacking any formal education in palliative care. It was also evident that ANs benefited from support from the team and especially from the RNs.

The participants described several difficult incidents when they felt alone. For example, they sometimes needed to make qualified assessments of complex care needs and decide when they would contact “experts,” despite being uncertain. These duties were beyond their qualifications and responsibilities. It was evident that the level of support for the ANs was suboptimal and they expected more support from RNs than they received. ANs expected that the RNs would step forward and take more responsibility when a situation became complicated. The feeling of isolation and the importance of multidisciplinary teamwork in palliative care for the older people has been described in other studies (Fryer, Bellamy, Morgan, & Gott, 2016; Phillips, Davidson, Jackson, & Kristjanson, 2008). The team is not only supportive in situations relating to the care of an individual patient but also acts as a body for learning and education. It is obvious that managers of RCFs must foster a more collaborative and team‐based work environment to improve the quality of palliative care. Many studies have highlighted the importance of teamwork in palliative care (Finucane, Stevenson, Moyes, Oxenham, & Murray, 2013; Fryer et al., 2016).

The scenarios described in our study were certainly a consequence of the structure of the consultant organization, under which an RN was located far away, was responsible for the care of many patients and could not be present at short notice. Thus, the consultant structure had the potential to affect the palliative care practices at an interpersonal level that may have had negative patient consequences, such as reduced quality of life during patients' last days. In fact, the practices of the consultant organization may directly contradict palliative care guidelines, especially when ANs' roles and relationships with RNs are varied and nonstandardized (Juthberg & Sundin, 2010; Reimer‐Kirkham et al., 2016). Previous studies have shown that ANs in RCFs are especially dependent on RN support in palliative care situations (Juthberg & Sundin, 2010; Karlsson, Ekman, & Fagerberg, 2008). In addition, studies have shown that access to RNs is key to safe, high‐quality care; thus, replacing RNs with ANs can have severe consequences for the quality of care (Aiken et al., 2012; Koy, Yunibhand, Angsuroch, & Fisher, 2017). Removing staff members who are well educated in palliative care from bedside care and creating an organization dependent on ANs with little education in palliative care who must then make important decisions when an RN is needed runs the risk of care failure and a lack of care consistency (Kane, Shamliyan, Mueller, Duval, & Wilt, 2007). Eventually, unmet expectations can lead to conflicts among staff members and potentially negatively affect care (Karlsson et al., 2008).

The second category, “presence and provision of psychosocial support,” reflects that the ANs perceived psychosocial support to be one of their most important tasks, which supports previous findings by Ahsberg and Carlsson (2014) that showed that ANs not only give practical palliative care but also engage in dialogue with patients on, for example, existential issues. The ANs expressed that psychosocial support was more complex and demanding when the patient reminded the participants of themselves or someone close to them because it led them to become too personally involved as they identified with the dying person. Becoming too involved at a personal and emotional level made it difficult for them to be present with the dying person. Palliative care situations affect professionals at an emotional and existential level that involves death and dying (Ahsberg & Carlsson, 2014; Beck, Jakobsson, et al., 2014; Jakobsson, Johnsson, Persson, & Gaston‐Johansson, 2006; Udo, Danielson, & Melin‐Johansson, 2013). Complexities in palliative care situations may arise suddenly and questions from the patient and family are not always planned, which makes it difficult for an AN to determine in advance when to involve the RN.

The ANs in this study expressed a tension between their commitment to ensure a peaceful death and bereavement for the family and the support that the organization could provide. ANs had personal ambitions to meet the patient's needs and give person‐centred care despite feeling alone and overloaded with responsibility. These results need to be taken seriously. Otherwise, the gap between the intended care and the practiced care of ANs may cause inner dilemmas, leading to stress as well as risking mental exhaustion (Gustafsson & Szebehely, 2005; Udo, Danielson, & Melin‐Johansson et al., 2013). In the long run, these inner conflicts can lead to burnout, high attrition rates, lack of consistency of care and, eventually, the departure of experienced caregivers from palliative care (Latta & Ross, 2010). It is well known that to give good care, staff members also need support and care (Armstrong & Daly, 2004). It has frequently been reported in international studies that possibilities for regular reflexive conversations about the difficult existential situations encountered in the care of severely ill and dying people is highly needed. This can strengthen self‐awareness in how to react in difficult situations and can strengthen the team (Finucane et al., 2013; Fryer et al., 2016).

Moreover, the results indicated that the delivery of palliative care was performed ad hoc, unstandardized and dependent on access to RNs. ANs stated that palliative care situations were influenced by the presence of clear care plans with agreed‐on goals for individual care. This observation is noteworthy because care that is dependent on ANs' individual skills and experiences without support from guidelines in the organization constitutes a vulnerable system that might threaten the quality of care. This system might also cause stress for the individual AN and raise the questions of whether laws and guidelines are being followed in practice and whether patient safety and high‐quality palliative care are truly being offered. The use of guidelines is a key factor that ensures the quality of palliative care (Lee, Bamford, Exley, & Robinson, 2015). Previously, Allen, O'Connor, Chapman, and Francis (2008) raised the question of whether palliative care guidelines in RCFs for older people are merely rhetoric or are indeed guiding care. Previous studies have also highlighted the importance of updated care plans with shared care goals for the person (Goodridge et al., 2005), which also contributes to care coordination in palliative care. Common goals also constitute a basis for high‐functioning teamwork (Beck‐Friis, 2008). A clear care plan may contribute to the strengthening of person‐centred palliative care (Silvester et al., 2013), especially in an organization with consultative status.

The last category, “the care environment as an important factor in palliative care,” demonstrates the features of the environment considered to be significant for the experience of palliative care. The environment helped the ANs to put patients at the centre of care and to create an atmosphere that supported dialogue. The ANs appreciated that the environment allowed them to have deeper conversations and dialogues with the patients. It is noteworthy that the ANs gave examples of features in the environment that often were not difficult or demanding to arrange, such as photos of the family. In contrast, they also said that negative experiences occurred when the room was noisy or too small for families to gather. The findings are supported by those of other studies that have shown that the physical environment is an important and meaningful factor in person‐centred practice (McCormack & McCance, 2006) and theoretical models proposing that person‐centred care in RCFs can be achieved by matching the environment with the capability of the individual (Lawton & Nahemow, 1973).

### 5.1 Study limitations

The interviews in this study reflect the participants' subjective, retrospective experiences and perceptions, not necessarily authentic facts. The participants were purposively selected and were a homogenous group; all of them were women born in a Scandinavian country. Consequently, there is a lack of ethnic diversity in the study sample. The data are limited to two municipalities in central Sweden and although there may be some possible transferability of the results to other municipalities in Sweden, the aim of this qualitative study was not to generalize results. Instead, this study may add further knowledge and deeper understanding about the important interaction between organizational structures and interpersonal palliative care. We found that the answers from the ANs followed the same pattern regardless of the RCFs where they worked, which strengthens the trustworthiness of the data despite the small number of respondents.

## 6 CONCLUSION

This study showed that ANs working in today's RCFs have a prominent function and role in providing palliative care at the end of life of an older person. In an organization where the RN is working on a consultant basis, there is a risk of care failure that needs to be evaluated. The staff closest to the patient in palliative care needs to have continuous education and support from the team, especially from the RN. It seems important to create a supportive environment for the AN to increase person‐centred care in palliative care in RCFs. The managers need to prioritize support and collaboration among staff members. As leaders in the patients' care and managers of the RCFs, RNs need to consider their own role in supporting, encouraging and supervising staff in palliative care. In a consultative organization in particular, there is a need for clear professional roles, collaborative care plans, structure and thorough and regular evaluations to ensure continuity of care.

## AUTHOR CONTRIBUTIONS

The last author (ME) supervised the study as a whole, including the development of the design, and preparation of the study. The second and third authors (MN and IS) collected the data, transcribed it, and conducted the data analysis under the supervision of the first author (CU), who evaluated the data analysis and was responsible for writing the manuscript. All authors critically revised the draft and have agreed on the final version.
